# Inequalities in access to paid sick leave among workers in England and Wales

**DOI:** 10.1002/hpm.3697

**Published:** 2023-08-07

**Authors:** Parth Patel, Sarah Beale, Vincent Nguyen, Isobel Braithwaite, Thomas E. Byrne, Wing Lam Erica Fong, Ellen Fragaszy, Cyril Geismar, Susan Hoskins, Annalan M. D. Navaratnam, Madhumita Shrotri, Jana Kovar, Anna Aryee, Andrew C. Hayward, Robert W. Aldridge

**Affiliations:** ^1^ Institute of Health Informatics University College London London UK; ^2^ Institute of Epidemiology and Health Care University College London London UK; ^3^ Department of Infectious Disease Epidemiology London School of Hygiene and Tropical Medicine London UK

**Keywords:** COVID‐19, health equity, health policy, public health

## Abstract

**Background:**

It is poorly understood which workers lack access to sick pay in England and Wales. This evidence gap has been of particular interest in the context of the Covid‐19 pandemic given the relationship between presenteeism and infectious disease transmission.

**Method:**

This cross‐sectional analysis (*n* = 8874) was nested within a large community cohort study based across England and Wales (Virus Watch). An online survey in February 2021 asked participants in work if they had access to paid sick leave. We used logistic regression to examine sociodemographic factors associated with lacking access to sick pay.

**Results:**

Only 66% (*n* = 5864) of participants reported access to sick pay. South Asian workers (adjusted odds ratio [OR] 1.40, 95% confidence interval [CI] 1.06–1.83) and those from Other minority ethnic backgrounds (OR 2.93, 95% CI 1.54–5.59) were more likely to lack access to sick pay compared to White British workers. Older workers (OR range 1.72 [1.53–1.93]–5.26 [4.42–6.26]), workers in low‐income households (OR 2.53, 95% CI 2.15–2.98) and those in transport, trade, and service occupations (OR range 2.03 [1.58–2.61]–5.29 [3.67–7.72]) were also more likely to lack access to sick pay compared respectively to workers aged 25–44, those in high income households and managerial occupations.

**Discussion:**

Unwarranted age and ethnic inequalities in sick pay access are suggestive of labour market discrimination. Occupational differences are also cause for concern. Policymakers should consider expanding access to sick pay to mitigate transmission of Covid‐19 and other endemic respiratory infections in the community, and in the context of pandemic preparation.

## 1 INTRODUCTION

An estimated two million employees in the UK do not earn enough to be eligible for statutory sick pay.^1^, ^2^ For those who do, mandatory paid sick leave as a proportion of previous earnings is among the lowest of the countries constituting the Organisation for Economic and Co‐operation and Development (OECD).^3^ Employees on casual or flexible contracts (including those on zero hour contracts) have a legal right to statutory sick pay if they are able prove their average earnings are above the eligibility threshold, which can be challenging. Statutory sick pay in the UK does not extend to those who are self‐employed.

There has been considerable policy attention to statutory sick pay in the context of the Covid‐19 pandemic.^2^, ^3^, ^4^, ^5^ From a living standards perspective, limited access to paid sick leave risked income loss and destitution for low income households during periods of Covid‐19 illness or self‐isolation. From a public health perspective, the risk of income loss may encourage presenteeism and drive transmission of Covid‐19 and other acute respiratory pathogens in the community. For example, fear of income loss was a common reason given for non‐adherence with self‐isolation among workers during the 2003 SARS outbreak in Toronto.^6^ Furthermore, the introduction of mandatory sick pay policies across US states has been associated with lower disease rates in the community and mitigation of flu epidemics.^7^, ^8^ Understanding factors associated with access to sick pay is consequently important to address both endemic Covid‐19 and respiratory viral transmission and for pandemic planning.

Official UK government estimates on sick pay coverage, which come from a 2014 survey of 2030 employees in Great Britain eligible for sick pay, found that 26% receive the statutory minimum rate, 57% receive sick pay above this minimum and 17% do not know.^9^ However, this survey does not capture features of workers lacking access to paid sick leave. The characteristics of that group can only indirectly be inferred by examining workers earning below the income threshold to access statutory sick pay using data from the Office of National Statistics' Labour Force Survey.^10^ This assumes all workers earnings above the eligibility threshold will automatically have access to paid sick leave. It is therefore limited in its ability to infer unwarranted labour market inequalities in access to paid sick leave.

Inequalities in access to sick pay among UK workers are poorly understood. In that context, this short communication exploits data from the Virus Watch community cohort study to examine factors associated with access to sick pay.

## 2 METHODS

### 2.1 Study design and procedure

Data were collected as part of the Virus Watch study, a prospective household cohort study of Covid‐19 transmission in England and Wales. The full study design and methodology has been described elsewhere.^11^ Participants were recruited using several methods including post, social media, SMS, and letters from General Practices. Eligibility criteria were consent to participate from all household members, household size up to 6 people (due to limitations of the online survey infrastructure), access to the internet and email, and at least one household member able to complete English‐language surveys.

After enrolling in the study, an initial baseline survey collected demographic, occupational, social and medical history data from participants. Monthly surveys collected detailed information on social, clinical and behavioural factors relevant to the phase of the pandemic as the time of collection. A February 2021 survey focussed on financial and work‐related determinants of COVID‐19 illness included an item relating to sick pay access. The analysis in this communication is limited to adults (>16) who responded to this February 2021 survey and reported being employed or self‐employed.

### 2.2 Exposures

The exposures of interest were demographic and social variables potentially associated with sick pay access among working adults. These were age; sex; region; ethnicity; household income; and occupation. Data on exposure variables were collected through the baseline survey completed on entry into the Virus Watch study.

### 2.3 Outcome

The outcome of interest was self‐reported access to sick pay. Participants were able to report ‘Yes’, ‘No’, ‘Unsure’ and ‘Not applicable’ when asked if they had access to paid sick leave if required. Those who responded ‘Not applicable’ were excluded from this study. Outcome data was collected between 17 and 28 February 2021.

### 2.4 Statistical analysis

To model the association between covariates and access to sick pay, we conducted univariable and multivariable fixed effects logistic regression models in R 4.0.3. A sensitivity analysis controlling for self‐employment status (employed vs. self‐employed) was also conducted.

### 2.5 Patient and public involvement

The study team worked with a community advisory group to inform equity‐related aspects of recruitment, design and dissemination. This advisory group, consisting of lay members of the public, community leaders, charities and policy experts, suggested this analysis.

## 3 RESULTS

Table 1 reports respondents' characteristics (*n* = 8874). Of respondents, 5864 (66%) reported having access to paid sick leave, 2218 (25%) reported no access to paid sick leave and 792 (8.9%) were unsure. Table 2 describes characteristics of those with and without access to sick pay.

**TABLE 1:** Sociodemographic characteristics of survey respondents.

Characteristic	*N* = 8874^a^
Age	
16–24	233 (2.6%)
25–44	2600 (29%)
45–64	5163 (58%)
65+	878 (9.9%)
Sex	
Female	4873 (55%)
Male	3980 (45%)
Missing	21 (0.2%)
Ethnicity	
White British	7487 (84%)
White Irish	138 (1.6%)
White other	657 (7.4%)
Black	53 (0.6%)
Mixed	131 (1.5%)
South Asian	259 (2.9%)
Other Asian	86 (1.0%)
Other ethnicity	40 (0.5%)
Prefer not to say	22 (0.2%)
Missing	1 (<0.1%)
Region	
East Midlands	779 (8.8%)
East of England	1862 (21%)
London	1482 (17%)
North East	390 (4.4%)
North West	962 (11%)
South East	1777 (20%)
South West	601 (6.8%)
Wales	167 (1.9%)
West Midlands	422 (4.8%)
Yorkshire and the Humber	378 (4.3%)
Missing	54 (0.6%)
Household income	
£0–24,999	1195 (13%)
£25,000–£49,999	2643 (30%)
£50,000–£74,999	2119 (24%)
£75,000+	2365 (27%)
Missing	552 (6.2%)
Employment status	
Employed	7294 (82%)
Self‐employed	1580 (18%)
Occupation	
Administrative & secretarial	1157 (13%)
Healthcare	628 (7.1%)
Indoor trades, process & plant	594 (6.7%)
Leisure & personal service	392 (4.4%)
Managers, directors & senior officials	637 (7.2%)
Missing	185 (2.1%)
Other professional & associate	3014 (34%)
Outdoor trades	202 (2.3%)
Sales & customer service	437 (4.9%)
Social care & community protective services	444 (5.0%)
Teaching, education & childcare	1008 (11%)
Transport & mobile machine	176 (2.0%)
Access to sick pay	
Yes	5864 (66%)
No	2218 (25%)
Unsure	792 (8.9%)

^a^ *n* (%).

**TABLE 2:** Description of participant characteristics by self‐reported access to sick pay.

	Access to sick pay		
Characteristic	Yes, *N* = 5864^a^	No, *N* = 2218^a^	Unsure, *N* = 792^a^
Age			
16–24	161 (69%)	39 (17%)	33 (14%)
25–44	2001 (77%)	408 (16%)	191 (7.3%)
45–64	3382 (66%)	1368 (26%)	413 (8.0%)
65+	320 (36%)	403 (46%)	155 (18%)
Sex			
Female	3358 (69%)	1098 (23%)	417 (8.6%)
Male	2491 (63%)	1115 (28%)	374 (9.4%)
Missing	15 (71%)	5 (24%)	1 (4.8%)
Ethnicity			
White British	4935 (66%)	1924 (26%)	628 (8.4%)
White Irish	88 (64%)	35 (25%)	15 (11%)
White other	457 (70%)	128 (19%)	72 (11%)
Black	35 (66%)	10 (19%)	8 (15%)
Mixed	92 (70%)	30 (23%)	9 (6.9%)
South Asian	168 (65%)	53 (20%)	38 (15%)
Other Asian	55 (64%)	17 (20%)	14 (16%)
Other ethnicity	19 (48%)	15 (38%)	6 (15%)
Prefer not to say	14 (64%)	6 (27%)	2 (9.1%)
Missing	1 (100%)	0 (0%)	0 (0%)
Region			
East Midlands	518 (66%)	202 (26%)	59 (7.6%)
East of England	1200 (64%)	509 (27%)	153 (8.2%)
London	994 (67%)	336 (23%)	152 (10%)
North East	280 (72%)	79 (20%)	31 (7.9%)
North West	672 (70%)	218 (23%)	72 (7.5%)
South East	1113 (63%)	480 (27%)	184 (10%)
South West	399 (66%)	162 (27%)	40 (6.7%)
Wales	113 (68%)	38 (23%)	16 (9.6%)
West Midlands	282 (67%)	100 (24%)	40 (9.5%)
Yorkshire and the Humber	256 (68%)	86 (23%)	36 (9.5%)
Missing	37 (69%)	8 (15%)	9 (17%)
Household income			
£0–24,999	586 (49%)	409 (34%)	200 (17%)
£25,000–£49,999	1676 (63%)	745 (28%)	222 (8.4%)
£50,000–£74,999	1525 (72%)	457 (22%)	137 (6.5%)
£75,000+	1757 (74%)	440 (19%)	168 (7.1%)
Missing	320 (58%)	167 (30%)	65 (12%)
Employment status			
Employed	5773 (79%)	954 (13%)	567 (7.8%)
Self‐employed	91 (5.8%)	1264 (80%)	225 (14%)
Occupation			
Administrative & secretarial	860 (74%)	200 (17%)	97 (8.4%)
Healthcare	422 (67%)	150 (24%)	56 (8.9%)
Indoor trades, process & plant	311 (52%)	222 (37%)	61 (10%)
Leisure & personal service	176 (45%)	162 (41%)	54 (14%)
Managers, directors & senior officials	462 (73%)	118 (19%)	57 (8.9%)
Missing	118 (64%)	50 (27%)	17 (9.2%)
Other professional & associate	2020 (67%)	743 (25%)	251 (8.3%)
Outdoor trades	55 (27%)	121 (60%)	26 (13%)
Sales & customer service	278 (64%)	116 (27%)	43 (9.8%)
Social care & community protective services	307 (69%)	104 (23%)	33 (7.4%)
Teaching, education & childcare	772 (77%)	163 (16%)	73 (7.2%)
Transport & mobile machine	83 (47%)	69 (39%)	24 (14%)

^a^ *n* (%).

Table 3 presents results of the univariable and multivariable logistic regression models. In the multivariable model, workers over the age of 65 (odds ratio [OR] 5.26, 95% CI 4.42–6.26) and between ages of 45 and 64 (OR 1.72, 95% CI 1.53–1.93) had greater odds of lacking access to sick pay in reference to workers aged 25–44. South Asian workers (OR 1.40, 95% CI 1.06–1.83) and ‘Other minority ethnic’ workers (OR 2.93, 95% CI 1.54–5.59) also had greater odds of lacking access to sick pay compared to White British workers. It is worth noting that although not always statistically significant, point estimates for workers from most minority ethnic backgrounds indicated elevated odds of lacking access to sick pay compared to White British workers. People in low income households were more likely to lack access to sick pay compared to those high income households, with households earning under £25,000 (OR 2.53, 95% CI 2.15–2.98) and households earning £25,000–£49,999 (OR 1.43, 95% CI 1.25–1.63) at greater odds of lacking access to sick pay if required than those in households earning above £75,000. Workers in leisure and personal service (OR 2.43, 95% CI 1.84–3.21), indoor trades, process and plant (OR 2.03, 95% CI 1.58–2.61), outdoor trades (OR 5.29, 95% CI 3.67–7.72) and transport and mobile machinery (OR 2.04, 95% CI 1.42–2.94) occupations were all more likely to lack access to sick pay compared to managers, directors and senior officials.

**TABLE 3:** Univariable and multivariable logistic regression models examining the relationship between sociodemographic characteristics and lacking access to sick pay.

	Univariable			Multivariable		
Characteristic	OR^a^	95% CI^a^	*p*‐value	OR^a^	95% CI^a^	*p*‐value
Age						
25–44	—	—		—	—	
16–24	1.49	1.11, 1.99	0.007	1.28	0.94, 1.74	0.11
45–64	1.76	1.58, 1.96	<0.001	1.72	1.53, 1.93	<0.001
65+	5.83	4.94, 6.88	<0.001	5.26	4.42, 6.26	<0.001
Sex						
Female	—	—		—	—	
Male	1.32	1.21, 1.45	<0.001	1.08	0.97, 1.19	0.2
Missing	0.89	0.32, 2.19	0.8	1.08	0.36, 2.84	0.9
Ethnicity						
White British	—	—		—	—	
White Irish	1.10	0.77, 1.55	0.6	1.20	0.82, 1.74	0.3
White other	0.85	0.71, 1.00	0.059	1.12	0.93, 1.35	0.2
South Asian	1.05	0.81, 1.35	0.7	1.40	1.06, 1.83	0.017
Other Asian	1.09	0.69, 1.68	0.7	1.25	0.77, 1.99	0.4
Black	0.99	0.55, 1.74	>0.9	0.99	0.53, 1.78	>0.9
Mixed	0.82	0.56, 1.19	0.3	1.04	0.69, 1.54	0.8
Other minority ethnicity	2.14	1.14, 4.02	0.017	2.93	1.54, 5.59	0.001
Prefer not to say	1.11	0.44, 2.58	0.8	1.11	0.43, 2.66	0.8
Household income						
£75,000+	—	—		—	—	
£0–24,999	3.00	2.60, 3.48	<0.001	2.53	2.15, 2.98	<0.001
£25,000–£49,999	1.67	1.48, 1.88	<0.001	1.43	1.25, 1.63	<0.001
£50,000–£74,999	1.13	0.99, 1.28	0.080	1.09	0.94, 1.25	0.2
Occupation						
Managers, directors & senior officials	—	—		—	—	
Administrative & secretarial	0.91	0.73, 1.14	0.4	0.70	0.56, 0.88	0.002
Healthcare	1.29	1.01, 1.64	0.039	1.13	0.88, 1.46	0.3
Indoor trades, process & plant	2.40	1.90, 3.05	<0.001	2.03	1.58, 2.61	<0.001
Leisure & personal service	3.24	2.49, 4.23	<0.001	2.43	1.84, 3.21	<0.001
Missing	1.50	1.06, 2.12	0.022	1.26	0.87, 1.80	0.2
Other professional & associate	1.30	1.08, 1.57	0.007	1.30	1.07, 1.59	0.009
Outdoor trades	7.06	4.97, 10.1	<0.001	5.29	3.67, 7.72	<0.001
Sales & customer service	1.51	1.16, 1.96	0.002	1.15	0.87, 1.52	0.3
Social care & community protective services	1.18	0.90, 1.54	0.2	0.85	0.64, 1.13	0.3
Teaching, education & childcare	0.81	0.64, 1.01	0.064	0.74	0.58, 0.94	0.013
Transport & mobile machine	2.96	2.10, 4.18	<0.001	2.04	1.42, 2.94	<0.001

Abbreviations: CI, confidence interval; OR, odds ratio.

^a^ OR >1 indicates increased odds of lacking access to sick pay.

A sensitivity analysis controlling for self‐employment status was consistent with these findings (Table 4). All observed differences in sick pay access between age, ethnic and income groups persisted. Indeed, ethnic contrasts in access to sick pay heightened. After adjusting for self‐employment status, 16–24‐year‐old workers (OR 1.73, 95% CI 1.25–2.38) also had greater odds of lacking access to sick pay compared to 24–44‐year‐old workers. With the exception of outdoor trade occupations, all observed occupational differences in sick pay access persisted.

**TABLE 4:** Sensitivity analysis multivariable logistic regression model including self‐employment status as a covariate.

Characteristic	OR^a^	95% CI^a^	*p*‐value
Age			
25–44	—	—	
16–24	1.74	1.25, 2.38	<0.001
45–64	1.29	1.13, 1.47	<0.001
65+	2.93	2.37, 3.62	<0.001
Sex			
Female	—	—	
Male	1.13	1.00, 1.28	0.057
Missing	1.06	0.28, 3.21	>0.9
Ethnicity			
White British	—	—	
Black	1.22	0.59, 2.35	0.6
Mixed	1.19	0.74, 1.87	0.5
Other Asian	1.25	0.70, 2.13	0.4
Other ethnicity	2.56	1.18, 5.35	0.014
Prefer not to say	1.54	0.55, 3.92	0.4
South Asian	1.87	1.38, 2.52	<0.001
White Irish	1.39	0.89, 2.12	0.14
White other	1.22	0.97, 1.52	0.078
Household income			
£75,000+	—	—	
£0–24,999	2.65	2.18, 3.22	<0.001
£25,000–£49,999	1.57	1.34, 1.85	<0.001
£50,000–£74,999	1.19	1.00, 1.41	0.049
Missing	1.66	1.29, 2.14	<0.001
Occupation			
Managers, directors & senior officials	—	—	
Administrative & secretarial	0.70	0.54, 0.91	0.007
Healthcare	0.72	0.53, 0.98	0.035
Indoor trades, process & plant	1.43	1.07, 1.92	0.016
Leisure & personal service	1.78	1.29, 2.45	<0.001
Missing	0.98	0.64, 1.49	>0.9
Other professional & associate	0.79	0.63, 1.00	0.049
Outdoor trades	1.30	0.79, 2.14	0.3
Sales & customer service	1.05	0.77, 1.43	0.8
Social care & community protective services	0.86	0.63, 1.18	0.4
Teaching, education & childcare	0.65	0.49, 0.85	0.002
Transport & mobile machine	1.79	1.18, 2.69	0.006

Abbreviations: CI, confidence interval; OR, odds ratio.

^a^ OR >1 indicates increased odds of lacking access to sick pay.

## 4 DISCUSSION

Our findings reveal substantial inequalities in access to paid sick leave among workers in England and Wales. Older workers, certain minority ethnic groups, workers in low‐income households and trade, transport and service occupations were more likely to lack access to paid sick leave. These differences were not explainable by self‐employment status.

This cross‐sectional analysis was nested in the larger Virus Watch prospective cohort study. By harnessing data already collected by this large cohort study, this analysis was able to fill an important policy‐relevant evidence gap on inequalities in sick pay access without needing to duplicate efforts to generate research data. Individuals in the Virus Watch study are well distributed across England and Wales and the cohort is diverse in terms of age, sex, ethnicity, and socioeconomic composition.^11^ Given participation in the Virus Watch study is voluntary and sampling non‐random, the cohort likely oversamples people concerned with COVID‐19 and participants were more likely to be White British, over the age of 65 and have a higher income than the general population. Our multivariable regression analysis adjusted for age, ethnicity and income to address this sampling bias, but any residual confounding is likely to mean our findings overestimate the true magnitude of age disparities in sick pay access and underestimate ethnic and income disparities in sick pay access.

There is little public data or previous literature on sick pay coverage in the UK. A descriptive analysis of 3974 UK workers found leisure and personal services, outdoor trades, transport and mobile machinery and indoor trades occupations have the lowest rates of paid sick leave coverage, corroborating the findings of our study.^12^ A 2014 survey of 2030 employees by the Department of Work and Pensions was limited to employees eligible to access sick pay.^9^ It reported descriptive differences in the amount of sick pay workers receive, with a greater proportion of older workers and those in leisure and personal service occupations reporting access to only the minimum statutory rate. There has been greater study of disparities in sick leave access in the US, where ethnic inequalities have been documented.^13^, ^14^

It is unsurprising that those in low‐income households are more likely to lack access to sick pay than those in high‐income households given statutory sick pay entitlement in the UK is conditional on earning above an income threshold. Inequalities in sick pay access between age and ethnic groups that cannot be explained by differences in income, occupation and employment status are suggestive of age‐ and race‐based discrimination in the labour market. Occupations with elevated odds of lacking access to sick pay relative to managerial occupations are classifiable as manual occupations and unskilled non‐manual occupations according to the widely used Goldthorpe class scheme.^15^ These occupations are referred to as *Working Class* in the Goldthorpe occupation‐based class taxonomy.^16^ That working class occupations are most likely to lack access to sick pay is cause for concern with regard to inequalities in the labour market.

In the context of the Covid‐19 pandemic, fear of income loss is likely to encourage presenteeism and SARS‐CoV‐2 transmission. A recent study of care homes in England found lower rates of SARS‐CoV‐2 transmission from staff when they had access to sick pay, compared to care homes where staff lacked access to statutory sick pay.^17^ Future research should investigate the relationship between access to sick pay and presenteeism in the community setting during the Covid‐19 pandemic.

More broadly, paid sick leave has been judged an effective intervention to reduce transmission of SARS‐CoV‐2 across OECD countries.^3^ The UK government made some changes to statutory sick pay when the pandemic began by allowing eligible employees to receive sick pay during periods of self‐isolation in addition to confirmed Covid‐19 illness, and to receive sick pay from the first day of illness or self‐isolation, rather than from the fourth day of illness as is the case for other illnesses. However, unlike around half of other OECD countries, the UK has not altered the wage replacement rate, nor has it modified the eligibility criteria to expand access to statutory sick pay. Given the inequalities we highlight in this paper, improving access to sick pay should be both an employment and health policy priority as Covid‐19 becomes an endemic disease and in the context of planning for future public health emergencies.

## CONFLICT OF INTEREST STATEMENT

AH serves on the UK New and Emerging Respiratory Virus Threats Advisory Group and was a member of the COVID‐19 transmission sub‐group of the Scientific Advisory Group for Emergencies. The other authors report no conflicts of interest.

## ETHICS STATEMENT

The Virus Watch study was approved by the Hampstead NHS Health Research Authority Ethics Committee: 20/HRA/2320, and conformed to the ethical standards set out in the Declaration of Helsinki. All participants provided informed consent for all aspects of the study.

## Data Availability

We aim to share aggregate data from this project as findings our website–https://ucl‐virus‐watch.net/. We also share some individual record level data on the Office of National Statistics Secure Research Service. Access to use of the data whilst research is being conducted is managed by the Chief Investigators (AH and RWA) in accordance with the principles set out in the UKRI guidance on best practice in the management of research data.
